# MeshGrow: Integrated framework for simulation-ready cardiac and vascular mesh construction from medical imaging

**DOI:** 10.1177/20480040261455944

**Published:** 2026-07-11

**Authors:** Numi Sveinsson Cepero, Arjun Narayanan, Fanwei Kong, Shawn C. Shadden

**Affiliations:** 1 Department of Mechanical Engineering, 1438 University of California, Berkeley, CA, USA; 2 Department of Mechanical Engineering and Materials Science, 7548 Washington University in St. Louis, St. Louis, MO, USA

**Keywords:** Cardiovascular modeling, cardiovascular simulation, deep learning, digital twin, medical image segmentation, patient-specific hemodynamics, computational fluid dynamics, machine learning-based mesh construction

## Abstract

Patient-specific cardiovascular simulations are an integral part of cardiovascular research and are increasingly finding use in clinical practice. A primary factor hindering large cohort studies and clinical utilization of cardiovascular simulations is the time-consuming process of constructing simulation-suitable computer models from medical image data. Methods have been proposed to automate the model construction process, but are tailored to either vascular or cardiac models. We herein propose a framework, MeshGrow, that is capable of combining automated modeling of cardiac and extended vascular territories. Namely, we demonstrate that this method can be used to reconstruct the main cardiac chambers along with the aorta and its main sub-branches, and returns a simulation-suitable mesh with a defined aortic valve surface and other surfaces needed for boundary condition specification. MeshGrow combines two different machine learning based techniques to address the specific challenges involved with cardiac and vascular model construction. This results in a two-stage approach; (a) meshing the cardiac structures and (b) growing the vasculature out from it. We present results of our method on five CT test datasets and compare predictions against state-of-the-art benchmark methods, as well as ground truth manually constructed models. Results show that MeshGrow achieves higher metric scores than benchmark methods on the test data. Additionally, we showcase the applicability of the method by running three-dimensional computational fluid dynamics simulations with two of the test cases. With this work, we demonstrate a method that can automate the construction of combined cardiac and vascular models for patient-specific cardiovascular hemodynamic simulation.

## Introduction

Patient-specific computational modeling of cardiovascular function is a fundamental part of cardiovascular research and increasingly a part of patient care.^1,2^ Specifically, patient-specific simulations of tissue mechanics and fluid dynamics gain insight into physiological function otherwise difficult, or impossible, to observe; for example, tissue mechanics, cardiac electrodynamics, and hemodynamic data such as pressure gradient or wall shear stress. These patient-specific simulations require anatomically accurate geometric models representing the domain of interest. These geometric models are typically built from medical image scans of patients, where the outlines of different tissues in the medical image (pixels/voxels) are used for reference for model construction. A primary challenge remains the time-consuming and costly process of geometric model construction from medical image data. This challenge limits the ability to consider larger cohort studies as well as timely decision support often needed in clinical settings.^1^ Also, because model construction is often manually performed and guided by user discretion, the process lacks standardization and the outcome may largely depend on the modeler.^3^

Several software tools have been developed for patient-specific cardiovascular modeling. Most of these, such as SimVascular and CRIMSON,^4,5^ focus on vascular modeling. The common workflow involves a user manually generating pathlines along vessel centers, followed by defining vessel lumen contours in planes perpendicular to these pathlines. These 2D contours are then lofted to construct a 3D surface mesh of the vasculature. This process heavily depends on manual input and user discretion, requiring experienced users to construct pathlines and refine 2D segmentations. Even with advancements in 2D segmentation techniques, such as level-set methods and machine learning,^6–8^ manual correction remains necessary. Additionally, selecting segmentation points along pathlines can lead to contour misalignment, complicating the 3D lofting process. Therefore, there is a clear need for accessible, automated vascular model construction methods.

Custom cardiac modeling software has not been developed to the same extent as vascular modeling tools. Most modelers rely on general medical image segmentation software such as 3D Slicer.^9^ Cardiac segmentation presents unique challenges due to the unclear boundaries between heart chambers in medical images. Moreover, temporal modeling introduces additional complexity, requiring multiple consistent cardiac models across different time points in a sequence. Prior methods have primarily used basic segmentation techniques, including manual voxel labeling and thresholding.^10^ While semi-automated approaches, such as level-set methods and region growing, offer potential improvements, they still require manual correction and parameter tuning.^10^

Recently, machine learning methods for segmentation have gained popularity as a possible avenue for automating the modeling process. For medical image segmentation, deep neural networks (DNNs) have shown promising results, especially for voxel-wise classification. Specifically, convolutional neural networks (CNNs) have been deployed for cardiac segmentation yielding good voxel classification accuracy but struggling with the resulting staircase artifacts and disconnected bodies, none of which is desirable when performing simulations.^11–13^

Alternative approaches, such as graph neural networks, have been used to deform mesh templates, improving topological consistency.^14^ However, this method introduces overlapping elements, which the recent method LinFlo-Net addresses by enforcing continuous and smooth deformations.^15^

For vascular segmentation, DNNs have been trained to classify voxels corresponding to coronary arteries, the aorta, and other structures.^16–18^ While these methods achieve high accuracy for large vessels with well-defined boundaries, they struggle with smaller branches, often resulting in disconnected structures. This limitation poses a significant challenge for simulation applications, where a unified mesh is required. SeqSeg addressed this issue by enhancing local vessel segmentation and prioritizing smaller branches.^19^ Beyond voxel classification, CNNs have also been applied to vascular tracking^20^ and bifurcation detection,^21^ but these approaches require integration with 2D or 3D lumen predictions to create complete vascular models.

To our knowledge, cardiac and vascular model construction methods have been developed largely independently. This is likely due to the historical separation of cardiac and vascular simulation applications. However, the physiology and pathology of the heart and its primary branches are closely interconnected. To address these applications, there is a need to integrate both cardiac and vascular components to produce comprehensive 3D models with well-defined intersection boundaries. We herein address this by developing a novel approach, MeshGrow. Namely, since vasculature and cardiac structure anatomies differ significantly topologically, we combine two different modeling approaches. First, we model the cardiac chambers by deforming a cardiac mesh template using LinFlo-Net,^15^ and from this we seed and grow out attached vasculature using SeqSeg.^19^ Overall, MeshGrow is capable of:

1. Automatically locating the cardiac region in a medical image volume and generating a patient-specific cardiac model.
2. Automatically locating the aortic root from the predicted cardiac model and initializing an automatic vascular tracking and segmentation method.
3. Automatically constructing an extensive vascular segmentation.
4. Returning a patient-specific cardiovascular mesh with an inlet, outlets and an aortic valve surface defined for boundary condition specification.

## Methods

**Figure 1.**
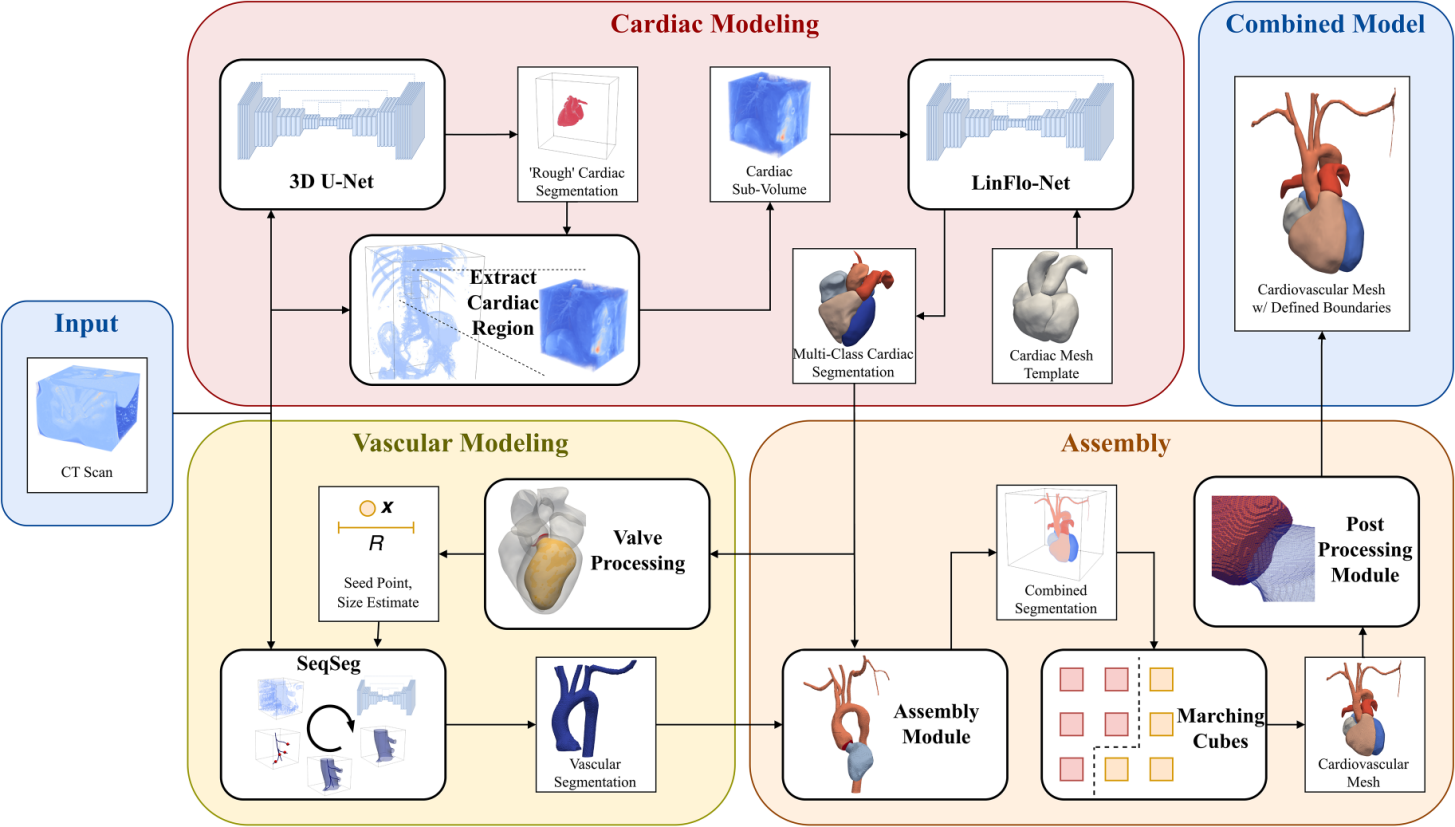
The MeshGrow method workflow. MeshGrow takes as input a medical image scan and returns a patient-specific cardiovascular mesh.

Figure 1 shows the method workflow. The input to the proposed method is a 3D angiography containing the heart and aorta. This “global” image volume is fed into a heart localization module that deploys a neural network to perform a “rough” cardiac segmentation. This segmentation is used to crop the global image volume to produce a smaller volume localized around the heart. This cardiac sub-volume is used for a detailed 8-class cardiac region segmentation. The 8 classes are: the left ventricular myocardium, left ventricle lumen, right ventricle lumen, left atrium lumen, right atrium lumen, ascending aortic arch lumen, pulmonary artery lumen, and aortic valve surface. The cardiac region segmentation is then used to place a seed point in the aortic root to initialize the segmentation of the aorta and its sub-branches. Note that the cardiac mesh template also contains a truncated ascending aortic arch lumen. As described later in this section, we use this to align the SeqSeg segmentation of the aorta and connecting arteries to the cardiac model. The cardiac and vascular segmentations are finally combined into one using an assembly module and output as surface meshes after post processing.

### Cardiac localization and segmentation

The first step is to locate the heart in the global image volume. We utilize a U-Net CNN for the task. Namely, we trained a model to perform binary voxel classification on the global image volume, where each voxel is labeled as either 1 (belonging to cardiac chambers) or 0 (belonging to any other tissue or organ). Specifically, voxels belonging to right or left ventricle or right or left atrium are labeled 1. We utilize the nnU-Net framework for hyperparameter selection and training.^22^ See Table 4 in Appendix for specifications of model architecture and data processing.

We use LinFlo-Net^15^ for cardiac mesh generation. This method transforms a template cardiac mesh to capture the patient-specific cardiac morphology as seen in a 3D clinical image (e.g. CT scan). LinFlo-Net is chosen for its demonstrated ability to predict accurate simulation-ready cardiac meshes. Briefly, the method utilizes a two-stage mesh deformation process consisting of a learned affine transformation (scaling, translation, and rotation) followed by a diffeomorphic mesh deformation process wherein the vertices of the template mesh are integrated along a learned static 3D flow vector field.

The affine transformation uses a 3D CNN and predicts the 9 parameters of the transformation (3 parameters each for scale, translation, rotation). This module is trained to minimize the chamfer distance in the L1-norm between the template mesh and target mesh. The diffeomorphic mesh deformation module consists of a U-Net architecture that is trained to produce a dense flow vector field in the image space. This module is trained to minimize a weighted sum of various losses including:

1. Chamfer distance in the L1-norm.
2. Normal consistency between template and ground truth meshes
3. Physics-based loss term that prevents volume collapse
4. Mesh regularization, which includes edge length, normal consistency across faces, and Laplacian smoothing loss.

The reader is referred to the original article^15^ for further details on each of these loss terms.

**Figure 2.**
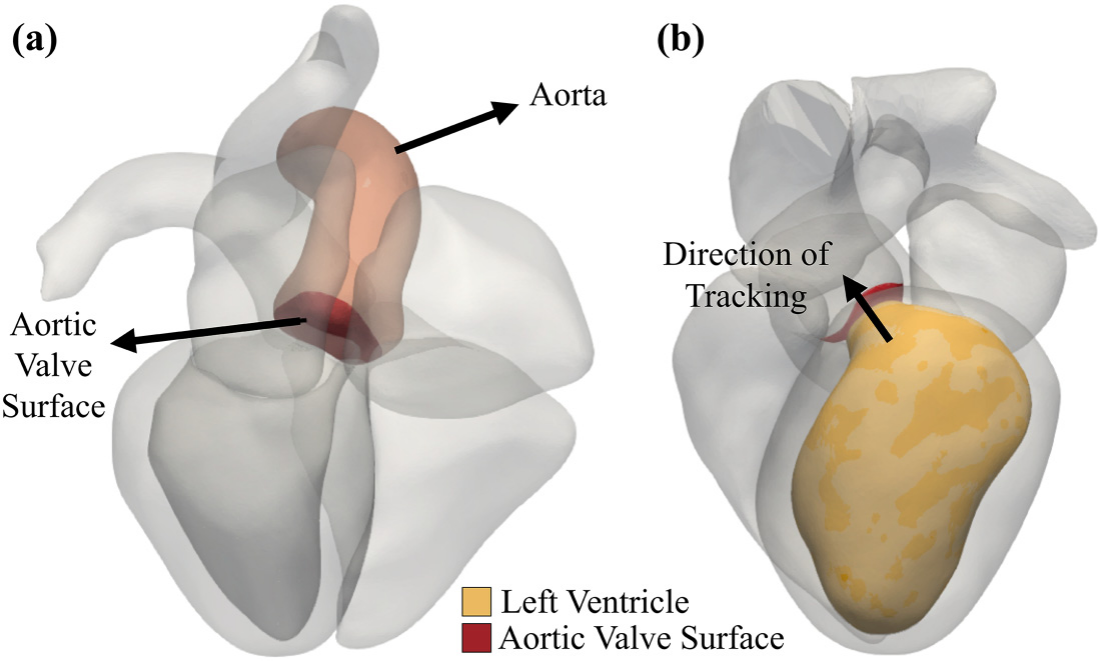
(a) Template mesh used for cardiac segmentation showing the added aortic valve surface. (b) Automatic initialization of aorta tracking starting from the aortic valve, determining direction using the aortic valve and left ventricle mesh regions.

The template mesh is a multi-component surface mesh representing the blood pools of the four cardiac chambers, the myocardium of the left ventricle, and segments of the pulmonary artery and aorta where they attach to the heart. Since the vascular segmentation method that we later use requires a seed-point for initialization, we added an additional region to the template mesh consisting of the aortic valve surface. Figure 2 shows the template mesh with the aortic root highlighted. This aortic valve surface in the template was created manually using MeshLab^23^ by cutting the aorta template mesh with a plane.

### Vascular segmentation

Vascular segmentation was achieved using the Sequential Segmentation (SeqSeg) method.^19^ The method constructs vessel segmentation by piece-wise addition of local segmentations predicted using a deep-learning-based U-Net convolutional neural network. SeqSeg automatically tracks vasculature by inferring direction and bifurcations from local vessel features. The method can be applied to various vascular territories, but requires initial specification of a seed point, tracking direction, and estimate of the initial local vessel radius.

SeqSeg deploys a DNN to perform vascular segmentation of local sub-volumes, including a 3D U-Net convolutional neural network with hyperparameters determined from the nnU-Net framework, see Isensee et al.^22^ for further details on methodology and Appendix Table 4 for specific hyperparameters chosen. For training we sampled each global vascular segmentation in the training dataset around the global vascular centerline. This improves use of available annotated data and introduces a beneficial inductive bias in our neural network model that allows it to generalize to vessels not annotated in training datasets, as demonstrated in Sveinsson Cepero and Shadden.^19^ Several augmentation techniques are used during training such as rotation, scaling, Gaussian noise, Gaussian blur, brightness, contrast, simulation of low resolution, gamma correction and mirroring; see Isensee et al.^22^ for full implementation details. We use a combined loss L of Dice score D(x,y) and binary cross entropy BCE(x,y) calculated between a pair of raw image data x and its ground truth binary vascular segmentation map y ; L(x,y)=1−D(x,y)+BCE(x,y) .

### Automatic initialization of vascular segmentation

We automatically initialize SeqSeg at the aortic root by processing the cardiac mesh prediction. Let us denote regions of mesh vertices as sets $S_{j}$ , where $S_{j}:=\{v_{i}|C(v_{i})=j\}$ where $v_{i}\in R^{3}$ is a vertex and C(v) is the cardiac class label map for j=1…8 . For initialization we use the regions representing left ventricle blood pool (LV), labeled j=3 and aortic valve surface (AV), labeled j=8 . Additionally, define corresponding sets $N_{j}$ of normal vectors $n_{i}\in R^{3}$ , $N_{j}:=\{n_{i}|C(v_{i})=j\}$ for each class j . For automatic initialization, these labeled regions are processed as follows:

1. The average node location is calculated for sets AV and LV; ${\bar{v}}_{j}=\frac{1}{|S_{j}|}\sum_{v_{i}\in S_{j}}v_{i},j\in [3,8]$ , and the average normal vector is calculated for AV; ${\bar{n}}_{8}=\frac{1}{|N_{8}|}\sum_{n_{i}\in N_{8}}n_{i}$ ,
2. There are two possible normals to the surface, the direction of tracking $\bar{d}\in R^{3}$ is chosen as the AV average normal ${\bar{n}}_{8}$ facing away from center of set LV ${\bar{v}}_{3}$
(1) $$\bar{d}=\left\{ \begin{array}{c} {\bar{n}}_{8},\;\mathrm{if}\;({\bar{v}}_{8}-{\bar{v}}_{3})\cdot {\bar{n}}_{8}>0\\ -{\bar{n}}_{8},\;otherwise \end{array}\right.$$

Initial seed point is chosen as D=2 radii distance into the aorta; $s_{0}={\bar{v}}_{8}+\bar{d}*D*R$ where R is aortic root radius estimate. We observed that only a rough radius estimate is enough for initialization; we thus used a fixed value of R=1.3cm .

### Tracking and assembly

Once initialized, SeqSeg extracts the first sub-volume around the aortic root for segmentation. A centerline is extracted from the segmentation, which is then used to determine direction and size of the vessel, and this information is used to choose the next point to move the segmentation window to. In the case of multiple branches, one point is chosen to move to next and the others are saved in a bifurcation queue for subsequent tracking, see Sveinsson Cepero and Shadden^19^ for further details. The global vascular segmentation is assembled together from all local segmentations predicted while tracking. Note that many of these local segmentations overlap and their probabilistic map output prediction are averaged together. We thresholded the averaged global probabilistic map using a value of 0.5 to get a binarized segmentation. A global vascular surface mesh was then created using marching cubes on the binarized segmentation, which was then smoothed, and a global centerline also extracted.

Outlets of the global vasculature mesh are detected using the global centerline. Where outlets are detected, caps are defined using a box clip boolean operation on the surface mesh to obtain a planar surface that can be used for boundary condition specification. Caps are planar surfaces defined at the cross section of vascular domain boundaries. The location of the node and the tangent of the centerline there are used to position and rotate the box clip operator. The box side length l is calculated according to l=3×R where R is the estimated radius of the vessel at that point, approximated from the local segmentation.

### Union of segmentations and valve definition

Two aortic segmentations are obtained; one from LinFlo-Net, $Y_{\mathrm{LF}}$ , and another from SeqSeg, $Y_{\mathrm{SS}}$ . We prioritize the $Y_{\mathrm{LF}}$ segmentation in a rectangular volume defined around the aortic valve to ensure correspondence to the predicted cardiac chambers and elsewhere we use $Y_{\mathrm{SS}}$ .

Let B denote a padded bounding box around the aortic valve surface, defined as the valve’s minimum axis–aligned bounding box enlarged by (L,W,H) voxels in the (x,y,z) directions. The final aortic segmentation $\hat{Y}$ is chosen voxel-wise as

(2) $$\hat{Y}(\;p)=\left\{ \begin{array}{c} Y_{\mathrm{LF}}(\;p),\;\text{if }\;p\in B,\\ \;Y_{\mathrm{SS}}(\;p),\;otherwise, \end{array}\right.$$

where (L,W,H)=(30,30,15) are the padding values applied to the bounding box in the respective spatial dimensions. Fewer voxels were padded to z direction because of image non-isotropic spacing, see Appendix Table 7 for exact size values for different image spacing. We pad the bounding box to ensure we capture the aortic valve surface, where only an approximate size is needed. Since the aortic valve is labeled in the LinFlo-Net prediction, this allows the aortic valve interface to be defined in the final combined model.

Once the final multiclass cardiovascular segmentation is defined with corresponding aortic segmentation $\hat{Y}$ , the marching cubes algorithm is applied to obtain a surface mesh representation of the geometric model.^24^ At interfaces where different segmentations border, we obtain vertices belonging to both meshed structures. This facilitates boundary condition specification for simulation. Finally, we perform global Laplace smoothing to get a smooth surface mesh ready for volumetric meshing and simulation. The end product is a global surface mesh with defined regions for different cardiac and vascular regions, as well as the aortic valve boundary and vascular caps.

### Experimental setup and metrics

We use the same data as Narayanan et al.^15^ to train LinFlo-Net. The training data includes the multi-modality whole heart segmentation challenge (MMWHS),^13^ orCaScore challenge,^25^ left atrial wall thickness challenge (SLAWT),^26^ and left-atrial segmentation challenge (LASC).^27^ In total we had 101 CT samples in our LinFlo-Net training dataset. We used the same data as Sveinsson Cepero and Shadden^19^ to train the neural network for SeqSeg, a total of 33 aortic models from the Vascular Model Repository (VMR, vascularmodel.com). See Sveinsson Cepero and Shadden^19^ for further details on training data.

Since the objective of this work is to present a unified method to generate consistent cardiac and vascular segmentations, it is important to consider image samples that capture the structures of interest sufficiently for testing. In particular, we are interested in cardiac and aortic segmentations, therefore we are interested in image samples that capture the whole heart along with the descending aorta and which include corresponding segmentation masks for comparative analysis. Datasets containing both cardiac and vascular segmentations are not readily available and, therefore, the authors had to partially manually construct the test dataset in-house, as described next.

Five CT test datasets that contained the heart, aorta and aortic branches, were selected from the VMR. See Appendix Table 5 for patient demographic details. These test samples were not included in training data. The image spacing ranged 0.31−0.58 mm in-plane and 0.62−1.25 mm out-of-plane, see Appendix Table 7. In-plane resolution was $512^{2}$ for all CT scans, with number of slices ranging 180−394 , see Appendix Table 7. Aortic coverage was from aortic root to descending aorta for all cases, with all except case 5 (O34421) containing parts of left subclavian artery, right subclavian artery, right common carotid and left common carotid arteries. Case 5 only covered parts of the brachiocephalic trunk as well as left subclavian artery and left common carotid artery.

These datasets already contained ground-truth segmentations of the vasculature to use to evaluate the predictive accuracy of MeshGrow, however, since the VMR only provides vascular segmentation, we had to generate the ground-truth cardiac segmentations. To do this, we used TotalSegmentator^28^ to generate the preliminary ground-truth cardiac segmentations that were then interrogated and manually corrected as needed. The segmentations generated directly from TotalSegmentator for all samples except O34421 appeared reasonable. This dataset required manual correction of the segmentation of the myocardium, as shown in Appendix Figure 7. Note, this particular failure case underscores the efficacy of a template-based method (which we adopt in MeshGrow) since mesh templates provide a strong shape prior for model predictions, enforcing topological consistency and anatomical accuracy.

For quantitative comparison between the segmentation results from our method, Y and the ground truth segmentations, X , we compute Dice score $D(X,Y)=\frac{2\cdot \|X\cap Y\|}{\left\|X\|+\|Y\right\|}$ . For aortic vascular segmentation results, we do a more comprehensive analysis and compute Hausdorff distance H(X,Y)=max{d(X,Y),d(Y,X)} where $\;d(X,Y)={\text{sup}}_{x\in X}{\text{inf}}_{y\in Y}$ d(x,y) and centerline overlap $CO(Y,C_{t})=\frac{\int_{C_{t}}Ydx}{\int_{C_{t}}dx}$ where $C_{t}$ is the ground truth centerline. Since our vascular segmentations are, in most cases, more extensive than the ground truth being compared to, we clip the prediction to place outlets at the same locations.

The cardiac segmentation method LinFlo-Net has been evaluated and compared with benchmarks in Narayanan et al.^15^ and therefore is not reconsidered here. However, since the vascular region segmentation depends on the cardiac segmentation method, it requires further validation. For comparison with state-of-the-art benchmark segmentation methods, as in Sveinsson Cepero and Shadden^19^ we compared against 2D and 3D nnU-Net segmentation predictions of the vascular segmentations on the test datasets. The benchmark models were trained on the same datasets as the method proposed. For full details on network architecture and data preprocessing, see Table 2 in Sveinsson Cepero and Shadden.^19^ Multiple augmentation techniques were deployed during training such as rotation, scaling, Gaussian noise and blur, see Supplementary Note 4 in Isensee et al.^22^ for full details.

**Table 1.** Quantitative metric results for aortic vascular regions.

Case	Name	D↑	H↓	CO↑
1	0174	0.972	0.412	0.984
2	0176	0.949	0.921	0.994
3	0188	0.953	1.065	0.964
4	O15032	0.957	0.668	0.988
5	O34421	0.964	0.443	0.961
**Avg.**	–	**0.959**	**0.702**	**0.978**
**Std.**	–	**0.008**	**0.258**	**0.013**

D , H (voxels), and CO are defined in Methods section. Avg.: average; std.: standard deviation.

Both benchmark and SeqSeg models were trained using stochastic gradient descent with Nesterov momentum. Initial learning rate of 0.01 was chosen with a learning rate scheduler, see Isensee et al.^22^ for full details. A single NVIDIA Geforce RTX 2080ti GPU (11 GB GPU memory) on the Savio High Performance Computing cluster at the University of California, Berkeley was used for training.

### Simulation

To demonstrate the applicability of our proposed unified cardiac and vascular modeling approach, we performed left ventricle-aorta hemodynamic simulations. We chose two arbitrary cardiovascular models in the test set. We isolated the left ventricle and aortic meshes for simulation. As these were single time point images, we required a way to predict cardiac motion over time. To do this, left ventricle contraction was mapped from realistic motion derived from a separate patient with gated CT. Namely, using our recent cardiac shape modeling approach,^29^ we first trained a neural ordinary differential equation (NODE) parameterized by time-series motion codes to fit the separate patient’s whole heart anatomy throughout the cardiac cycle. We then trained a second NODE to learn the diffeomorphic transformation between this patient’s anatomy at the static time point and the cardiovascular anatomies of our test cases. The learned cardiac motion from the first NODE (described by the motion codes) was subsequently mapped onto the test cases to generate their motion over the cardiac cycle. We performed a 3D blood flow computational fluid dynamics simulation using svFSI,^30^ where the arbitrary Lagrangian and Eulerian (ALE) formulation of the incompressible Navier–Stokes equations was solved using the finite element method. We used the synthetic left ventricle and aorta motion to prescribe velocity boundary conditions on the left ventricle and aorta wall, and used a linear elastostatic model to solve the motion of the entire computational domain. We prescribed zero pressure boundary conditions at vessel cap outlets for simplicity. We simulated systole where the aortic valve is open. We obtain simulation results for velocity, pressure and wall shear stress over time.

## Results

### Geometric

We tested the proposed method on five withheld CT test cases. Appendix Tables 1 and 2 show the quantitative metric results for our method on these test cases, vascular and cardiac, respectively.

**Table 2.** Quantitative metric results for cardiac chambers of the geometric model.

	Case	Name	MYO	LA	LV	RA	RV	PA	AO
D↑	1	0174	0.866	0.915	0.955	0.916	0.943	0.856	0.964
	2	0176	0.806	0.814	0.909	0.891	0.913	0.692	0.953
	3	0188	0.831	0.934	0.940	0.823	0.840	0.852	0.957
	4	O15032	0.880	0.937	0.959	0.927	0.926	0.678	0.949
	5	O34421	0.861	0.862	0.937	0.866	0.892	0.893	0.972
	**Avg.**	–	**0.849**	**0.892**	**0.940**	**0.885**	**0.903**	**0.794**	**0.959**
	**Std.**	–	**0.027**	**0.048**	**0.018**	**0.037**	**0.036**	**0.090**	**0.008**
	Case	Name	MYO	LA	LV	RA	RV	PA	AO
H↓	1	0174	5.663	3.950	4.547	6.049	7.234	61.467	7.684
	2	0176	11.512	13.365	9.696	11.068	9.190	58.498	35.473
	3	0188	9.925	6.056	5.899	11.472	12.140	62.595	10.491
	4	O15032	8.379	4.148	3.936	6.058	7.246	60.035	31.448
	5	O34421	11.481	12.976	12.137	12.510	8.924	63.157	7.449
	**Avg.**	–	**9.392**	**8.099**	**7.243**	**9.431**	**8.947**	**61.150**	**18.509**
	**Std.**	–	**2.194**	**4.207**	**3.162**	**2.798**	**1.793**	**1.703**	**12.321**

Cardiac chambers include MYO : myocardium, LA : left atrium, LV : left ventricle, RA : right atrium, RV : right ventricle, PA : pulmonary artery, and AO : aorta. D and H are defined in Methods section. Avg.: average; std.: standard deviation.

**Figure 3.**
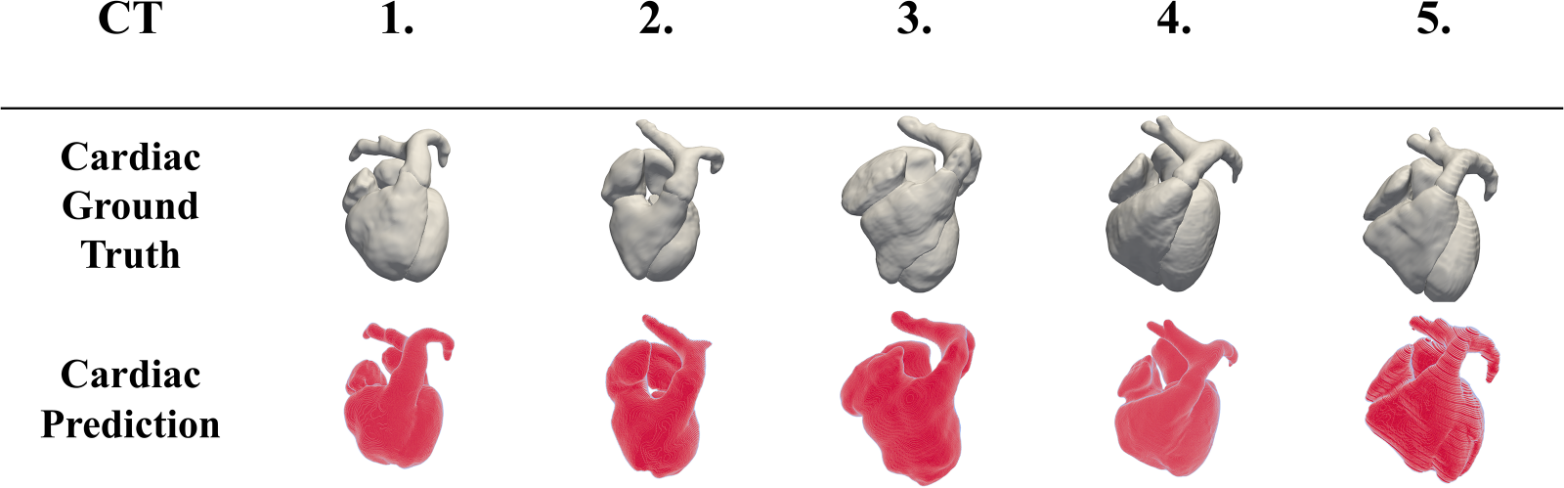
Comparing the ‘‘rough’’ binary cardiac segmentations with the ground truth for the CT test set.

The method begins by performing a ‘‘rough’’ binary cardiac segmentation in the global image volume to estimate the location and bounds of the cardiac structures. See Figure 3 for qualitative comparison of the binary segmentation with ground truth cardiac models for the five held out CT test cases.

**Figure 4.**
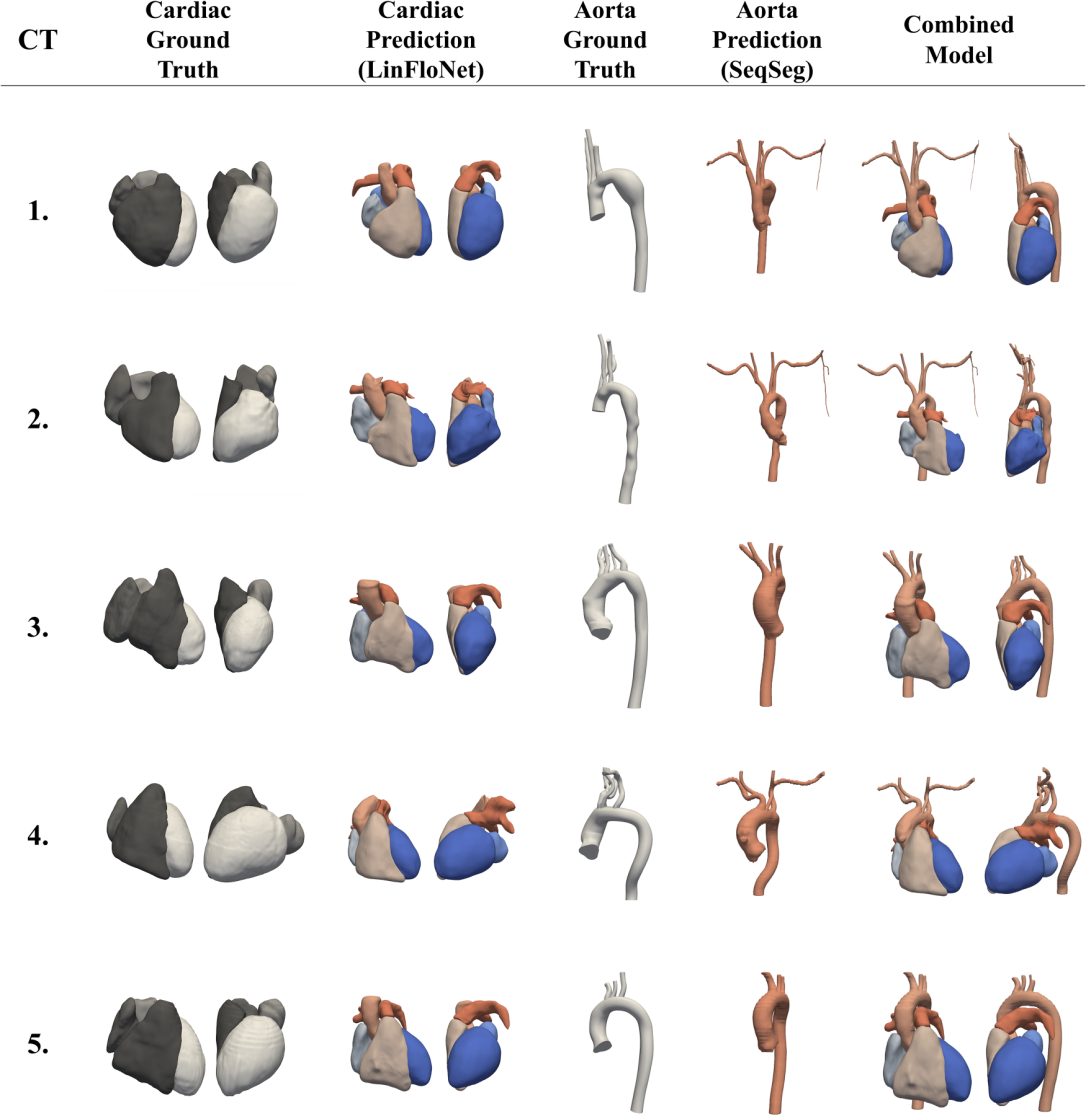
Qualitative results for our method compared to ground truth segmentations. From left: cardiac region ground truth, cardiac region prediction results (from LinFlo-Net), aorta ground truth, aorta prediction results (from SeqSeg), and the final combined cardiovascular model.

After localizing the cardiac structures and extracting a sub-volume around its bounds, the method predicts a multiclass cardiac model. The Dice score and Hausdorff distance results for the different cardiac regions are presented in Appendix Table 2 for the 5 test datasets. The average Dice score for each region is shown in bold font. Of the cardiac structures, the Dice score was highest for the ventricles; on average 0.940±0.018 and 0.903±0.036 , for the left and right, respectively. On average, the Dice score for atrium predictions were 0.892±0.048 and 0.885±0.037 for the left and right, respectively. The Hausdorff distance results show most cardiac structures achieving scores in the range of 7−10 voxels on average, except for the vascular regions where the pulmonary artery and aorta get 61.15±1.70 and 18.51±12.32 voxels, respectively. The resulting meshes are presented for comparison with ground truth models in the first two columns in Figure 4.

The seed point for initiating aortic segmentation is determined from the aortic root predicted in the cardiac model. To test the robustness of seed point size to initialize SeqSeg tracking, we try a range of radii values 0.8−1.8 cm, for which normal aortic root size typically ranges.^31^ Results are presented in Appendix Table 6, where initialization works for all sizes except for 1.8 cm where it failed for one case, indicating SeqSeg robustness to different seed point size values. To test the method robustness to seed location, the displacement factor D was perturbed to range 1−3 with Appendix Table 6 showing successful SeqSeg initialization for all values.

Appendix Figure 6 shows the vascular segmentation growing from the cardiac mesh for case 2. The quantitative metrics comparing the predictions with the ground-truth for the five CT test cases are presented in Appendix Table 1. On average, our method achieved a Dice score of 0.959±0.008 , Hausdorff distance of 0.702±0.258 (in voxels), and centerline overlap of 0.978±0.013 . For a qualitative comparison, the predicted vascular segmentations and the ground truth models are shown in Figure 4, third and fourth columns. The resulting cardiac and vascular segmentations combined into unified cardiovascular models are shown in the last column of Figure 4.

For the comparison with vascular segmentation using the state-of-the-art 2D and 3D nnU-Net benchmark method,^22^ quantitative metric results are presented in Appendix Table 3. On average, the benchmark methods achieve Dice score of 0.91−0.92 , Hausdorff distance of 1.68−1.89 voxels and centerline overlap score of 0.94−0.95 . Our method outperformed the benchmark for vascular segmentations for all metrics presented.

**Table 3.** Quantitative comparisons for aortic vascular region test set CT cases between state-of-the-art 2D nnU-Net and 3D nnU-Net segmentation methods using the dice score ( D ), Hausdorff distance ( H , voxels), and centerline overlap ( CO ).

		D↑		H↓		CO↑	
Case	Name	2D U-Net	3D U-Net	2D U-Net	3D U-Net	2D U-Net	3D U-Net
1	0174	0.923	0.901	1.281	1.976	0.951	0.939
2	0176	0.845	0.865	1.544	1.804	0.916	0.992
3	0188	0.947	0.946	0.991	0.999	0.951	0.942
4	O15032	0.951	0.938	3.237	3.216	0.911	0.864
5	O34421	0.934	0.932	1.358	1.473	1.000	1.000
**Avg.**	–	**0.920**	**0.916**	**1.682**	**1.894**	**0.946**	**0.947**
**Std.**	–	**0.039**	**0.030**	**0.798**	**0.740**	**0.032**	**0.049**

Avg.: average; std.: standard deviation.

**Table 4.** U-Net architecture and training specifications for the nnU-Net models.

**CT (SeqSeg)**	
Intensity Normalization	0.5/99.5% clip + foreground z-score
Image Spacing	0.200, 0.0547, 0.0547
Target Spacing	0.200, 0.0547, 0.0547
Patch Size	[20, 80, 80]
Batch Size	33
Max Nr. Features	320
Nr. Stages Encoder	5
Nr. Stages Decoder	4
Nr. Layers per Stage	2
Nr. Pooling Ops. per Axis	[2, 4, 4]
Conv. Kernel Size	[3, 3, 3]
**CT (Cardiac)**	
Intensity Normalization	0.5/99.5% clip + foreground z-score
Image Spacing	1.5, 1.5, 1.5
Target Spacing	1.5, 1.5, 1.5
Patch Size	[192, 112, 112]
Batch Size	2
Max Nr. Features	320
Nr. Stages Encoder	6
Nr. Stages Decoder	5
Nr. Layers per Stage	2
Nr. Pooling Ops. per Axis	[5, 4, 4]
Conv. Kernel Size	[3, 3, 3]

For a runtime comparison of methods, we additionally report approximate inference times. The MMWHS paper^13^ claims that it takes 6 − 10 hours for a complete manual cardiac segmentation. Inference time for LinFlo-Net is under 10s per sample running on CPU. SeqSeg inference takes 20 − 40 minutes for aortic tree vascular segmentation compared to 8 − 10 hours for a manual segmentation.^19^ Additionally, SeqSeg runs automatically whereas manual model construction requires manual input from an expert during runtime.

### Simulation

**Figure 5.**
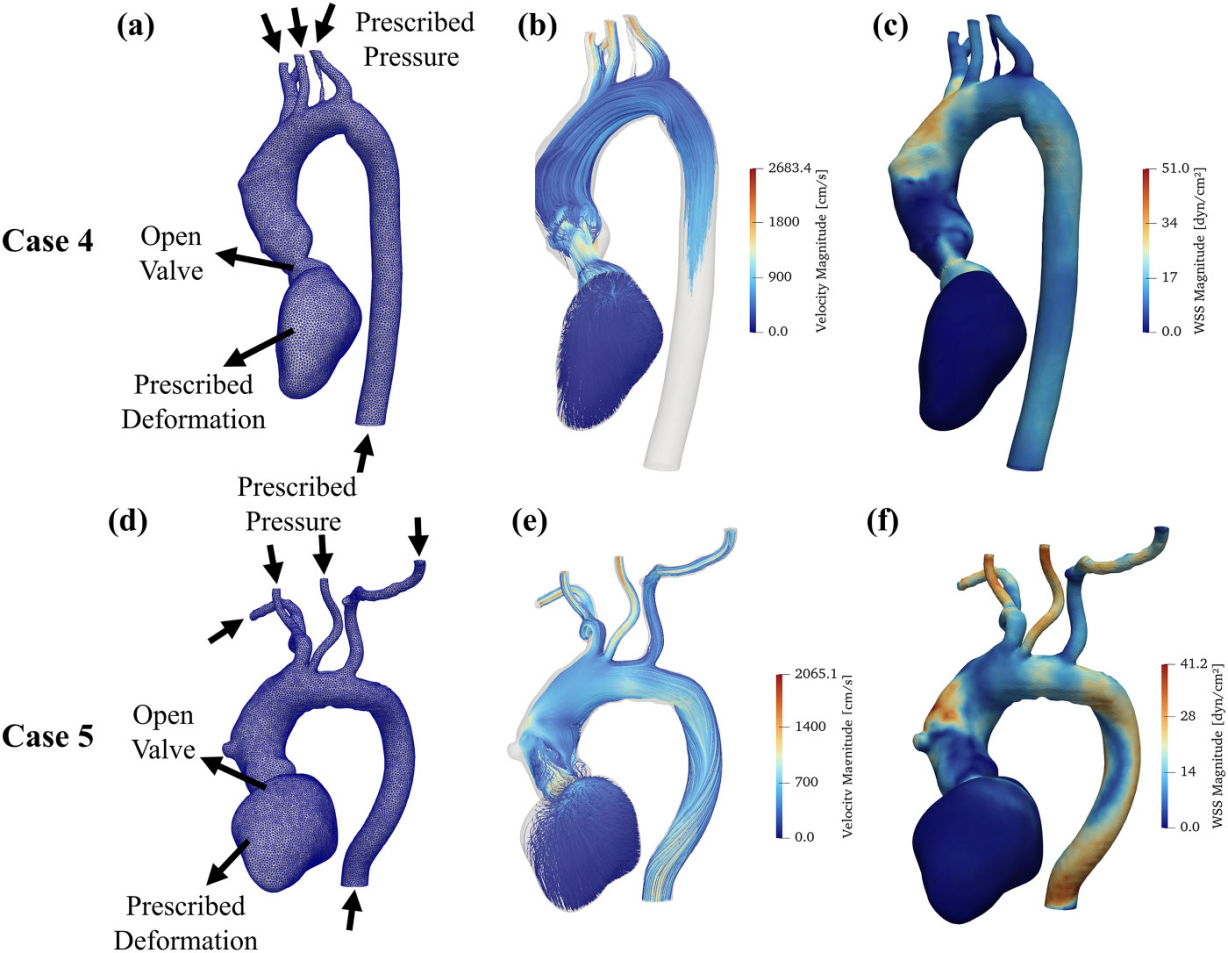
Hemodynamic simulation results using surface meshes acquired using MeshGrow (cases 4 and 5 in Appendix Tables 1 to 3). Deformation boundary condition was prescribed on LV with pressure prescribed at outlets and an open aortic valve (a,d). Streamlines passing through aortic valve (color coded for velocity magnitude) (b,e) and wall shear stress (WSS) magnitude (c,f) are shown during systole.

To demonstrate the method’s ability to produce simulation-ready models, we ran hemodynamic simulations for two arbitrary cases in the test set. Figure 5 shows simulation results for cases 4 and 5. We display streamlines that pass through the aortic valve and wall shear stress magnitude during systole.

**Figure 6.**
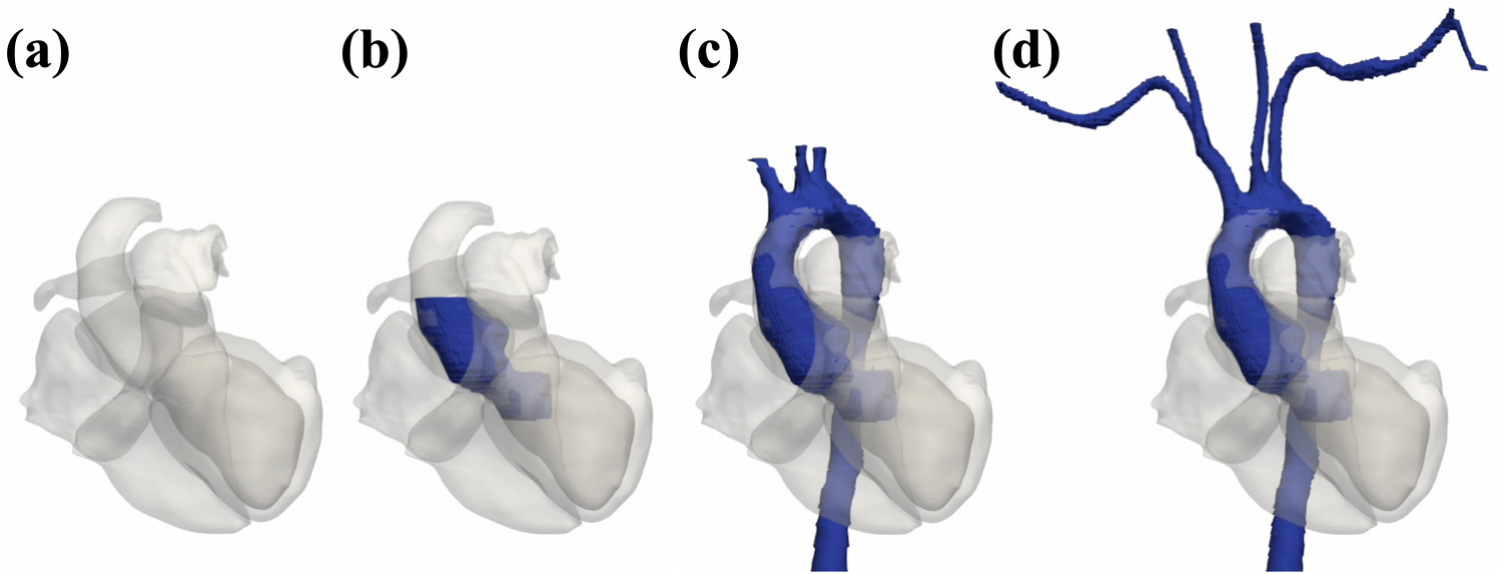
Seed for SeqSeg placed in the aortic root of the predicted cardiac mesh. SeqSeg takes steps to grow the aortic segmentation out from the cardiac mesh. Displayed are the cardiac mesh (a), and final aortic segmentation (d) as well as two intermediate states (b,c).

**Figure 7.**
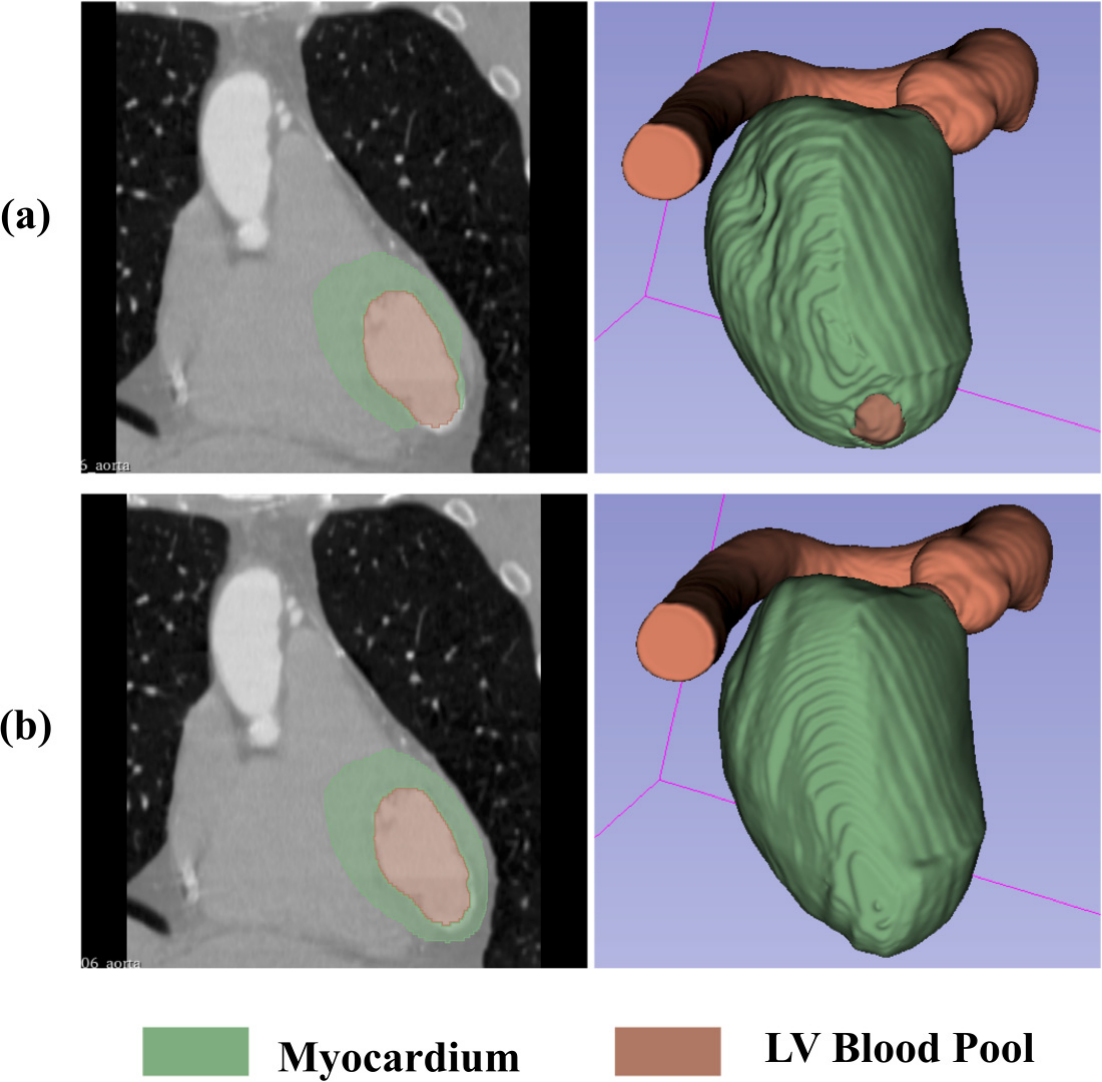
(a) Cardiac segmentations of case 5 (ID O34421) produced by TotalSegmentator contain a hole at the apex of the myocardium. (b) We manually fixed this segmentation for evaluation purposes.

## Discussion

We presented a novel automatic cardiovascular model construction method. The method takes in a medical image scan of a patient as input and outputs a patient-specific three-dimensional simulation-ready mesh of the cardiac chambers and the aorta with defined inlets, outlets and aortic valve interface. This is, to our knowledge, the first combined automatic cardiac *and* vascular model construction method.

Two independent deep-learning-based modeling approaches for cardiac and vascular model construction were used. Both approaches require neural network training. We trained and tested the method on a dataset of CT image scans. We compared the predictions on held-out test datasets to the corresponding manually constructed 3D models. The cardiac chamber segmentations achieved, on average, Dice scores of 0.85±0.03 , 0.89±0.05 , 0.94±0.02 , 0.89±0.04 , and 0.90±0.04 for the myocardium, left atrium, left ventricle, right atrium, and right ventricle, respectively. Because of the nature of SeqSeg being a step-wise growth approach, the vascular segmentations often extend further than the ground truth cap placements. After placing caps at the same locations, the vascular segmentations, on average, obtained a Dice score of 0.96±0.01 , Hausdorff distance score of 0.70±0.26 voxels, and centerline overlap score of 0.98±0.01 . We compared the vascular segmentation results against the state-of-the-art nnU-Net method and showed that the proposed method outperformed 2D and 3D benchmarks for all metrics considered.

Inspired by the template-deformation approaches of Kong, Narayanan, and Shadden,^14,15^ we adopted the LinFlo-Net paradigm for predicting cardiac chambers. For predicting vasculature, however, the greater topological variability makes template deformation less suitable, so we instead used the step-based vessel-tracking approach of SeqSeg. In this work, we focused on modeling of aortic vasculature for LV-aortic simulations. In future work, we hope that SeqSeg can similarly be applied to modeling of the pulmonary arteries or other connected vasculature.

Because the downstream vascular structures originate from well-defined valve locations, tracking methods that require seed points, such as SeqSeg, become straightforward to deploy. Rather than searching the full image for vascular roots, seed points can be placed directly from the predicted cardiac model. As presented in Appendix Table 6, SeqSeg initialization is robust to seed size and placement, making initialization even simpler.

A primary limitation of this work is the size of the test dataset. No public dataset containing both cardiac and vascular ground truth models currently exists, which required us to build our own and constrained its scale. We hope this work will be expanded to the evaluation of additional image sets, and also the inclusion of other image types, namely MR. Further assessment of the robustness of SeqSeg’s automatic initialization is also needed, including experiments that incorporate segmentation errors. Lastly, future work could explore how these machine-learning-based modeling approaches affect downstream simulation results.

## Conclusion

We present an automated method to construct three-dimensional geometric models of the heart and aortic vasculature from medical imaging. We hope this work can facilitate patient-specific cardiovascular simulation research and its further entry into clinical settings for patient care. Future directions can include investigating the effects of machine learning based automated model construction methods on simulation results as well as incorporating additional anatomical features, for example, pulmonary arteries, valve models, and veins.

## Appendix

**Table 5.** Patient demographics and hemodynamic data for the included study.

ID	Dx	Age	Sex	Wt	Ht	HR	BP
0174	MFS	18	M	58.4	183.5	75	100/64
0176	MFS	25	M	77	193	125	104/59
O34421	SVGD	71.5 ± 9.5	F/M 1/13	–	–	–	–
O15032	SVGD	71.5 ± 9.5	F/M 1/13	–	–	–	–
0188	CAD	50	F	–	–	62	–

*Abbreviations*: ID = model identifier; dx = diagnosis (MFS: Marfan syndrome, H: healthy, SVGD: saphenous vein graft disease, CAD: coronary artery disease); age = years; sex (F/M) = female/male; wt = weight (kg); ht = height (cm); HR = heart rate (beats per minute); BP = systolic/diastolic cuff blood pressure (mmHg). Entries marked “–” indicate unavailable metadata. Note: O34421 and O15032 are distinct models from the same publication, and their reported patient demographics are identical aggregate statistics.

**Table 6.** Robustness to seed point size and placement in aortic root as described in Method section.

Seed size ( R )	0174	0176	0188	O15032	O34421
1.8 cm	✓	✓	✓	✓	X
1.6 cm	✓	✓	✓	✓	✓
1.4 cm	✓	✓	✓	✓	✓
1.3 cm	✓	✓	✓	✓	✓
1.2 cm	✓	✓	✓	✓	✓
1.0 cm	✓	✓	✓	✓	✓
0.8 cm	✓	✓	✓	✓	✓
Seed distance ( D )					
1.0	✓	✓	✓	✓	✓
1.5	✓	✓	✓	✓	✓
2.0	✓	✓	✓	✓	✓
2.5	✓	✓	✓	✓	✓
3.0	✓	✓	✓	✓	✓

Automatic initialization of vascular segmentation. Showing whether SeqSeg initializes correctly and tracks down the aorta using different radius values for each patient and a displacement for direction.

**Table 7.** Test dataset CT image details and physical dimensions of a 30×30×15 pixel patch (in cm).

	0176	0174	0188	O15032	O34421
Total Nr. slices	249	344	394	367	180
In-plane res. (cm)	0.058	0.057	0.031	0.039	0.038
Out-of-plane res. (cm)	0.1245	0.080	0.062	0.062	0.123
x (cm)	1.74	1.71	0.9375	1.17	1.14
y (cm)	1.74	1.71	0.9375	1.17	1.14
z (cm)	1.8675	1.20	0.93	0.93	1.845
